# The cytochrome P450 *CYP6P4* is responsible for the high pyrethroid resistance in *knockdown resistance*-free *Anopheles arabiensis*

**DOI:** 10.1016/j.ibmb.2015.10.015

**Published:** 2016-01

**Authors:** Sulaiman S. Ibrahim, Jacob M. Riveron, Robert Stott, Helen Irving, Charles S. Wondji

**Affiliations:** a Vector Biology Department, Liverpool School of Tropical Medicine, Liverpool, L3 5QA, United Kingdom; b Department of Health and Social Sciences, Leeds Beckett University, LS1 3HE, Leeds, United Kingdom

**Keywords:** *Anopheles arabiensis*, Pyrethroids resistance, Metabolic, *CYP6P4*, δ-ALA, δ-aminolevulinic acid, *An*., Anopheles, cDNA, complementary DNA, CYPED, cytochrome P450 Engineering Database, DDT, dichlorodiphenyltrichloroethane, DDE, dichlorodiphenyldichloroethylene, IPTG, Isopropyl β-d-1-thiogalactopyranoside, NADP, nicotinamide adenine dinucleotide phosphate, ompA, outer membrane protein A, P450cam, P450 camphor hydroxylase, PLANTS_PLP_, *Piece-wise linear potential* Protein-Ligand ANT System, qRT-PCR, quantitative reverse transcriptase-polymerase chain reaction, *Rdl*, resistance to dieldrin, *rp1*, resistance to pyrethroids 1

## Abstract

Pyrethroid insecticides are the front line vector control tools used in bed nets to reduce malaria transmission and its burden. However, resistance in major vectors such as *Anopheles arabiensis* is posing a serious challenge to the success of malaria control.

Herein, we elucidated the molecular and biochemical basis of pyrethroid resistance in a *knockdown resistance*-free *Anopheles arabiensis* population from Chad, Central Africa. Using heterologous expression of P450s in *Escherichia coli* coupled with metabolism assays we established that the over-expressed P450 *CYP6P4,* located in the major pyrethroid resistance (*rp1*) quantitative trait locus (QTL), is responsible for resistance to Type I and Type II pyrethroid insecticides, with the exception of deltamethrin, in correlation with field resistance profile. However, *CYP6P4* exhibited no metabolic activity towards non-pyrethroid insecticides, including DDT, bendiocarb, propoxur and malathion. Combining fluorescent probes inhibition assays with molecular docking simulation, we established that *CYP6P4* can bind deltamethrin but cannot metabolise it. This is possibly due to steric hindrance because of the large vdW radius of bromine atoms of the dihalovinyl group of deltamethrin which docks into the heme catalytic centre.

The establishment of *CYP6P4* as a partial pyrethroid resistance gene explained the observed field resistance to permethrin, and its inability to metabolise deltamethrin probably explained the high mortality from deltamethrin exposure in the field populations of this Sudano-Sahelian *An. arabiensis*. These findings describe the heterogeneity in resistance towards insecticides, even from the same class, highlighting the need to thoroughly understand the molecular basis of resistance before implementing resistance management/control tools.

## Highlights

• *An. arabiensis* is a major malaria vector in Sudano-Sahelian Africa.
• Functional characterisation established *CYP6P4* as a key pyrethroid resistance gene in *An. arabiensis.*
• *CYP6P4* metabolizes pyrethroids, including permethrin, bifenthrin and λ-cyhalothrin.
• *However,* the P450 does not metabolise deltamethrin, though inhibition assay revealed that the P450 binds deltamethrin.
• *In silico* analyses revealed that in contrast to other pyrethroids, deltamethrin binds unproductively in *CYP6P4.*

## Graphical abstract

**Figure:**
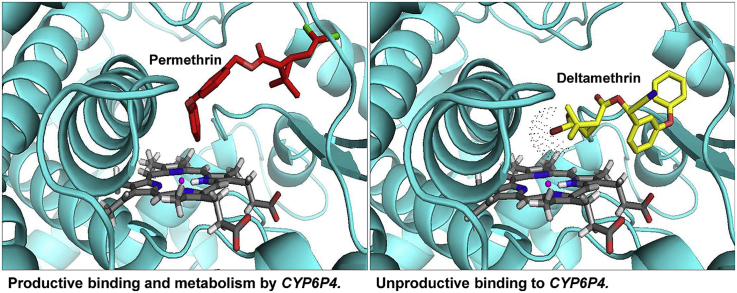

## 1 Introduction

Within the last ten years the burden of malaria has been greatly reduced in sub-Saharan Africa thanks to the scale-up of pyrethroid-impregnated long-lasting insecticidal treated nets (LLINs) (WHO, 2014) and indoor residual spraying (IRS) (WHO, 2006). By 2013, these interventions, in addition to chemotherapy, have helped reduce malaria-related mortality in the WHO African region by 54% (WHO, 2014). However, the disease still claimed 584,000 lives in 2013 alone, 90% of which occurred in the WHO African region (WHO, 2014). One of the challenges threatening these malaria intervention tools is the widespread resistance to the major insecticides used in LLINs and IRS, notably from the *Anopheles gambiae* complex and *Anopheles funestus* group (Coetzee and Koekemoer, 2013, Corbel and N'Guessan, 2013).

Across Africa, resistance to insecticides is heterogeneous even some times over relatively small distances (Ranson et al., 2009), thus implementation of any resistance management demands sound knowledge of dominant vector species distribution, behaviours, insecticide susceptibility/resistance status, and most importantly the molecular mechanisms of the resistance (Coetzee et al., 2000, Corbel and N'Guessan, 2013, Gatton et al., 2013).

In insects, two major mechanisms of resistance to insecticides have been described: (i) metabolic resistance due to over-expression and/or increase in the activity of the major enzymes involved in detoxification of insecticides (Hemingway and Ranson, 2000), and (ii) target-site insensitivity, which results in decreased sensitivity of the molecular target of the insecticide through point mutations, as in the voltage-gated sodium channel (*kdr* mutations), acetylcholinesterase (*ace-1* mutation) or the gamma-amino butyric acid mutation (*Rdl* mutation) (Du et al., 2005, Ffrench-Constant et al., 2000, Martinez-Torres et al., 1998, Ranson et al., 2000, Weill et al., 2004, Wondji et al., 2011). Recent evidence has stressed the preeminent role of metabolic resistance as the most important mechanism of resistance in the major Anopheline mosquito vectors (Hemingway, 2014) with cytochrome P450s especially from the CYP6 family taking the front seat in conferring resistance to the four major insecticides used for public health interventions (Duangkaew et al., 2011, Edi et al., 2014, Riveron et al., 2014, Riveron et al., 2013).

Besides *An. gambiae*, *An. arabiensis* is the most efficient malarial parasite vector of the *An. gambiae* complex (Gilles and De Meillon, 1968) and in some places, especially the drier savannah, it remains the dominant vector species. Prospect of control of *An. arabiensis* through exploitation of indoor resting and feeding behaviours is confounded by its marked plastic behaviours, including marked zoophily, exophily and exophagy (Coetzee et al., 2000, Durnez and Coosemans, 2013, Sinka et al., 2011). There is also growing concern over the great role *An. arabiensis* is playing in residual malaria transmission even in settings where robust malaria control tools are effectively implemented (Durnez and Coosemans, 2013, Killeen, 2014). *An. arabiensis* is the dominant vector species in Chad, Central Africa where it's reported to be resistant to pyrethroids but susceptible to the carbamate bendiocarb and organophosphates, malathion and fenitrothion (Kerah-Hinzoumbe et al., 2008, Ranson et al., 2009, Witzig et al., 2013).

In 2009, the *An. arabiensis* populations from Ndjamena (*Ndja*), Chad were resistant to permethrin (Type I pyrethroid) and DDT (an organochlorine), only moderately resistant to a Type II pyrethroid deltamethrin (90% mortality rate), but susceptible to bendiocarb (a carbamate), malathion (an organophosphate) and dieldrin (an organochlorine) (Witzig et al., 2013). No 1014F or 1014S *kdr* mutations were detected in the *Ndja* population and the PBO synergist assay fully restored susceptibility to pyrethroids, suggesting metabolic resistance as the cause of pyrethroid resistance. Witzig and colleagues identified a major pyrethroid resistance QTL (*rp1*) in the 2R chromosomal arm which alone explained a quarter of the genetic variance to permethrin resistance. The QTL was enriched in P450s, some of which are orthologs of genes implicated in pyrethroid resistance in *An. gambiae* and *An. funestus* (Edi et al., 2014, Wondji et al., 2009). A qRT-PCR analysis revealed one of the CYP genes (*CYP6P4*) to be up-regulated 22-fold in the permethrin resistant populations compared with the susceptible populations. The role of this P450 in conferring metabolic resistance to *An. arabiensis* was also recently pointed out in a population from neighbouring Sudan, where microarray-based transcription profiling detected *CYP6P4* as one of the most over-expressed detoxification genes (Abdalla et al., 2014). However, there is so far no functional evidence that the *An. arabiensis CYP6P4* is responsible for the metabolic resistance toward the pyrethroids. In case *CYP6P4* is playing a role it remains unknown as to why the same population exhibited only a moderate resistance to deltamethrin. It also becomes imperative to establish whether *CYP6P4* is a cross-resistance gene, able to confer both Type I pyrethroid and DDT resistance.

To fill these gaps in knowledge, we performed a functional characterisation of the *CYP6P4*, establishing that it is the major P450 responsible for pyrethroid resistance in the *kdr*-free population of *An. arabiensis* from Chad. Using a combination of heterologous expression and *in vitro* characterisation we demonstrated the role of this P450 in metabolism of Type I and Type II pyrethroids with the exception of deltamethrin. Combining homology modelling and molecular docking simulations we established why this P450 could not metabolize deltamethrin, dissecting the molecular basis of deltamethrin susceptibility in these Chadian *An. arabiensis* populations.

## 2 Methods

### 2.1 Mosquito samples

The mosquitoes used in this research were adult, female *An. arabiensis*, field collected from Ndjamena (12° 6ʹ N, 15° 2ʹ E) by Witzig and colleagues (Witzig et al., 2013) and established as the *Ndja* colony in the Liverpool School of Tropical Medicine, UK. The populations were confirmed as *An. arabiensis* species using PCR (Scott et al., 1993). Susceptibility status, biochemical assays, QTL mapping and analysis of the expression pattern of the P450s spanning the *rp1* QTL of this population are given in detail in the above publication (Witzig et al., 2013).

### 2.2 Amplification and cloning of full length cDNA of An. arabiensis CYP6P4

RNA was extracted using the PicoPure RNA isolation Kit (Arcturus, Applied Biosystems, USA) from three pools of 10 permethrin-resistant female mosquitoes from *Ndja* as described in (Witzig et al., 2013). cDNA was synthesized from extracted RNA using SuperScript III (Invitrogen, USA) with oligo-dT20 and RNAse H (New England Biolabs, USA). Full length coding sequences of *CYP6P4* were amplified separately from cDNA of 10 mosquitoes using the Hot Start II Taq Polymerase (Thermo Fisher, UK) and the primers in Supplementary Table S1. To 14 μl PCR mix made up of 5× Phusion HF Buffer (with 1.5 mM MgCl_2_ in final reaction), 85.7 μM dNTP mixes, 0.34 μM each of forward and reverse primers, 0.015U of Phusion High-Fidelity DNA Polymerase (Fermentas, Massachusetts, USA) and 10.71 μl of dH_2_0, 1 μl cDNA was added. Amplification was carried out using the following conditions: one cycle at 95 °C for 5 mins; 35 cycles of 94 °C for 20s (denaturation), 57 °C for 30s (annealing), and extension at 72 °C for 90s; and one cycle at 72 °C for 5 mins (final elongation).

PCR products were cleaned individually with QIAquick^®^ PCR Purification Kit (QIAGEN, Hilden, Germany) and cloned into pJET1.2/blunt cloning vector using the CloneJET PCR Cloning Kit (Fermentas). These were then cloned into the *Escherichia coli DH5α,* plasmids miniprepped with the QIAprep^®^ Spin Miniprep Kit (QIAGEN) and sequenced on both strands using the above primers.

### 2.3 Cloning and heterologous expression of recombinant *An. arabiensis* CYP6P4 in *E. coli*

The pJET1.2 plasmid bearing the full length coding sequence of *CYP6P4* was utilised to prepare the gene for expression. *CYP6P4* was prepared by fusing cDNA fragment from a bacterial *ompA+2* leader sequence with its downstream ala–pro linker to the NH_2_-terminus of its cDNA, in frame with its initiation codon, as described (Pritchard et al., 1997). This is achieved in a PCR reaction using the primers given in Table S1. Details of these PCRs are provided in previous publications (Riveron et al., 2014). The PCR product was cleaned, digested with *Nde*I and *Xba*I restriction enzymes and ligated into the expression vector pCWori + already linearized with the same restriction enzymes to create expression plasmid pB13:*ompA+2*-*CYP6P4.* This plasmid was co-transformed together with a plasmid bearing *An. gambiae* cytochrome P450 reductase (pACYC-AgCPR) into *E. coli JM109*. Membrane expression and preparation follows the procedure as described previously (Pritchard et al., 2006). Recombinant CYP6P4 expressed optimally at 21 °C and 150 rpm 36 h after incubation following induction with 1 mM IPTG and 0.5 mM δ-ALA to the final concentration. Membrane content of the P450 and cytochrome P450 reductase activity were determined as established (Omura and Sato, 1964, Strobel and Dignam, 1978).

### 2.4 In vitro metabolism assays with insecticides

Metabolism assays were conducted with pyrethroids (Types I and II), the pseudo-pyrethroid etofenprox, DDT, bendiocarb and propoxur, as well as malathion, using protocols as previously described (Riveron et al., 2014, Stevenson et al., 2011). 0.2 M Tris–HCl and NADPH-regeneration components were added to the bottom of 1.5 ml tube chilled on ice. Membranes expressing recombinant CYP6P4 and *Ag*CPR (and reconstituted with cytochrome b_5_) were added to the side of the tube and pre-incubated for 5 min at 30 °C, with shaking at 1200 rpm. 20 μM of test insecticide was then added into the final volume of 0.2 ml (∼2.5% v/v methanol), and reaction started by vortexing at 1200 rpm and 30 °C for 1 h. Reactions were quenched with 0.1 ml ice-cold methanol and incubated for 5 more minutes to precipitate protein. Tubes were then centrifuged at 16,000 rpm and 4 °C for 15 min, and 150 μl of supernatant transferred into HPLC vials for analysis. All reactions were carried out in triplicate with experimental samples (+NADPH) and negative controls (-NADPH). For pyrethroids, 100 μl of sample was loaded onto an isocratic mobile phase (90:10 v/v methanol to water) with a flow rate of 1 ml/min, monitoring wavelength of 226 nm and peaks separated with a 250 mm C18 column (Acclaim ™ 120, Dionex) on Agilent 1260 Infinity at 23 °C. For DDT 1 mM sodium cholate (solubilising agent) was added as described previously (Mitchell et al., 2012) and absorption monitored at 232 nm. For the other insecticides, mobile phases were 65:35 (bendiocarb) and 60:40 v/v (propoxur and malathion) acetonitrile to water, respectively with column temperature of 40 °C for bendiocarb and propoxur. Bendiocarb and propoxur were detected at 205 nm and 270 nm respectively, whilst malathion was detected at 230 nm. Enzyme activity was calculated as percentage depletion (the difference in the amount of insecticide(s) remaining in the +NADPH tubes compared with the –NADPH) and a t-test used for statistical analysis.

Kinetic analysis was conducted with permethrin by measuring the rate of reaction under linear conditions for 30 min while varying the substrate concentrations (3μM-30μM) in presence of 22.5 pmol recombinant proteins. Reactions were performed in triplicates both for + NADPH (experimental tubes) and –NADPH (negative control tubes). *K*_*m*_ and *V*_*max*_ were established from the plot of substrate concentrations against the initial velocities and fitting of the data to the Michaelis–Menten module using the least squares non-linear regression, as implemented in the GraphPad Prism 6.03 Software (GraphPad Inc., La Jolla, CA, USA).

### 2.5 Fluorogenic probes assay

To find out whether there are differences in the degree of binding of recombinant CYP6P4 to different insecticides, membrane was also tested for O-dealkylation towards seven (7) fluorogenic probes: resorufin-based 7-ethoxyresurofin (7-ER), resurofin methyl ether (RME), resorufin-benzylether (RBE) and resorufin-pentylether (RPE); coumarin-based probes: 7-ethoxy-4-trifluoromethylcoumarin (7-EFC) and 7-methoxy-4-trifluoromethylcoumarin (MFC); and diethoxyfluorescein (DEF). In a total volume of 225 μl containing ∼10 pmol CYP6P4, buffered with 50 mM potassium phosphate buffer (KPi at pH 7.4 with 5 mM MgCl_2_), 1 μM probe substrate was added. Membranes were activated for 5 min at 37 °C in fluorescence spectrophotometer FLOUstar Omega (BMG LABTECH, Ortenberg, Germany) before 25 μl NADPH regeneration buffer (1 mM glucose-6-phosphate (G6P), 0.25 mM MgCl_2_, 0.1 mM NADP and 1 U/ml glucose-6-phosphate dehydrogenase (G6PDH) prepared in KPi (pH 7.4) was added. All reactions were conducted in three replicates with negative control (–NADPH) devoid of the regeneration buffer. Rate of fluorescent product formation for 7-ER, RBE and RME (ʎ_exc_ = 544 nm, ʎ_emi_ = 590 nm), 7-EFC and MFC (ʎ_exc_ = 410 nm, ʎ_emi_ = 535 nm) and DEF (ʎ_exc_ = 485 nm, ʎ_emi_ = 530 nm) was determined by linear regression of measurement between 2 and 10 min after start of the reaction.

For kinetics, 0–2 μM diethoxyfluorescein was assayed with 2 pmol of recombinant enzyme in a total volume of 250 μl. The protocol was as outlined above, with varying substrate concentration. Incubation was done under conditions established to be linear with respect to time. Steady-state kinetic parameters were obtained by measuring the rate of reaction for 10 min while varying the substrate concentration from 0 to 2 μM. *K*_*m*_ and *V*_*max*_ were established from the plot of substrate concentrations against the initial velocities through a non-linear regression, by fitting the data to the Michaelis-Menten equation using GraphPad Prism 6.03 (GraphPad Software Inc., La Jolla, CA, USA).

To assess differences in the degree of binding of the recombinant CYP6P4 to insecticides from different classes, inhibition assay was conducted with DEF and nine insecticides (inhibitors). Miconazole, a potent P450s inhibitor (Lupetti et al., 2002) was utilised as a positive control inhibitor. IC_50_ determination was conducted as described in previous studies (Bambal and Bloomer, 2006, Kajbaf et al., 2011). In a total volume of 225 μl buffered with 50 mM KPi (pH 7.4), containing 0.11 μM DEF (∼*K*_*m*_ values), 2 pmol CYP6P4, 25 μM test inhibitors or miconazole was spiked in the top wells and serially diluted into eight-fold concentrations (25–0.011 μM). 25 μl of pre-warmed regeneration buffer (7.8 mg glucose-6-phosphate, 0.25 mM MgCl_2_, 1.7 mg NADP, 6 U/ml glucose-6-phosphate dehydrogenase and 2% w/v NaHCO_3_) was incorporated into the wells. Fluorescence was monitored for 21 cycles at interval of 1 min with shaking at every step, at 30 °C. Results were analysed with GraphPad Prism software and inhibition at each inhibitor concentration and incubation time calculated as residual control activity towards DEF.

### 2.6 Sequence characterisation of An. arabiensis CYP6P4

In order to identify the characteristic features of *CYP6P4* which can impact its monooxygenase activity, putative substrate recognition sites 1–6 of *An. arabiensis CYP6P4*, *An. gambiae CYP6P4*, and *An. funestus CYP6P4a* and *CYP6P4b* were determined by mapping their amino acid sequences to that of *Pseudomonas putida CYP101A* (P450cam) (Gotoh, 1992, Poulos et al., 1985). In addition, structurally conserved regions were also predicted using the CYPED tool (Sirim et al., 2010).

### 2.7 Homology modelling and molecular docking simulation

To predict the pattern of molecular interaction of *CYP6P4* with the substrate insecticides, a 3D model of this P450 was created using MODELLER *9v2* (Fiser and Sali, 2003) and CYP3A4 as a template (PDB: 1TQN) (Yano et al., 2004) with overall 35% identity. Virtual datasets of ligand insecticides: 1*R*-*cis* permethrin (ZINC01850374), bifenthrin (ZINC02516821), deltamethrin (ZINC01997854), λ-cyhalothrin (ZINC01843672), etofenprox (ZINC02558051), DDT (ZINC01530011), bendiocarb (ZINC02015426) and malathion (ZINC01530799), were retrieved from the library in ZINC^12^ database (https://zinc.docking.org/) (Irwin and Shoichet, 2005). Docking simulations were carried out using the *CLC bio* Drug Discovery Workbench 2.0 (http://www.clcbio.com/products/clc-drug-discovery-workbench/) with binding site set as a sphere centred above the heme iron and covering 20 Å radius. For each ligand, 50 binding poses were generated and sorted according to hybrid PLANTS_PLP_ score (Korb et al., 2009) and conformation in the protein's active site. Figures were prepared using the PyMOL 1.7 (DeLano WL, 2004) and Molegro Molecular Viewer *2.5* (http://www.clcbio.com/).

## 3 Results

### 3.1 Expression pattern of recombinant CYP6P4

Recombinant CYP6P4 expressed optimally between 36 and 40 h after induction with average concentration of 8.18 nmol/ml ± 2.06 (n = 3) and P450 content of 1.24 nmol/mg protein ± 0.36 (n = 3). This is higher than the concentration reported for recombinant CYP6P3 and CYP6M2 from *Anopheles gambiae* (Muller et al., 2008, Stevenson et al., 2011). The cytochrome *c* reduction assay produced an activity of 92.04 cytochrome *c* reduced/min/mg protein ± 10.70 (n = 3) of the cytochrome P450 reductase, higher than obtained from co-expression of CYP6P3 from *An. gambiae*, but lower than obtained from co-expression with CYP6M2 (Muller et al., 2008, Stevenson et al., 2011).

### 3.2 An. arabiensis CYP6P4 metabolism of pyrethroids

**Fig. 1:**
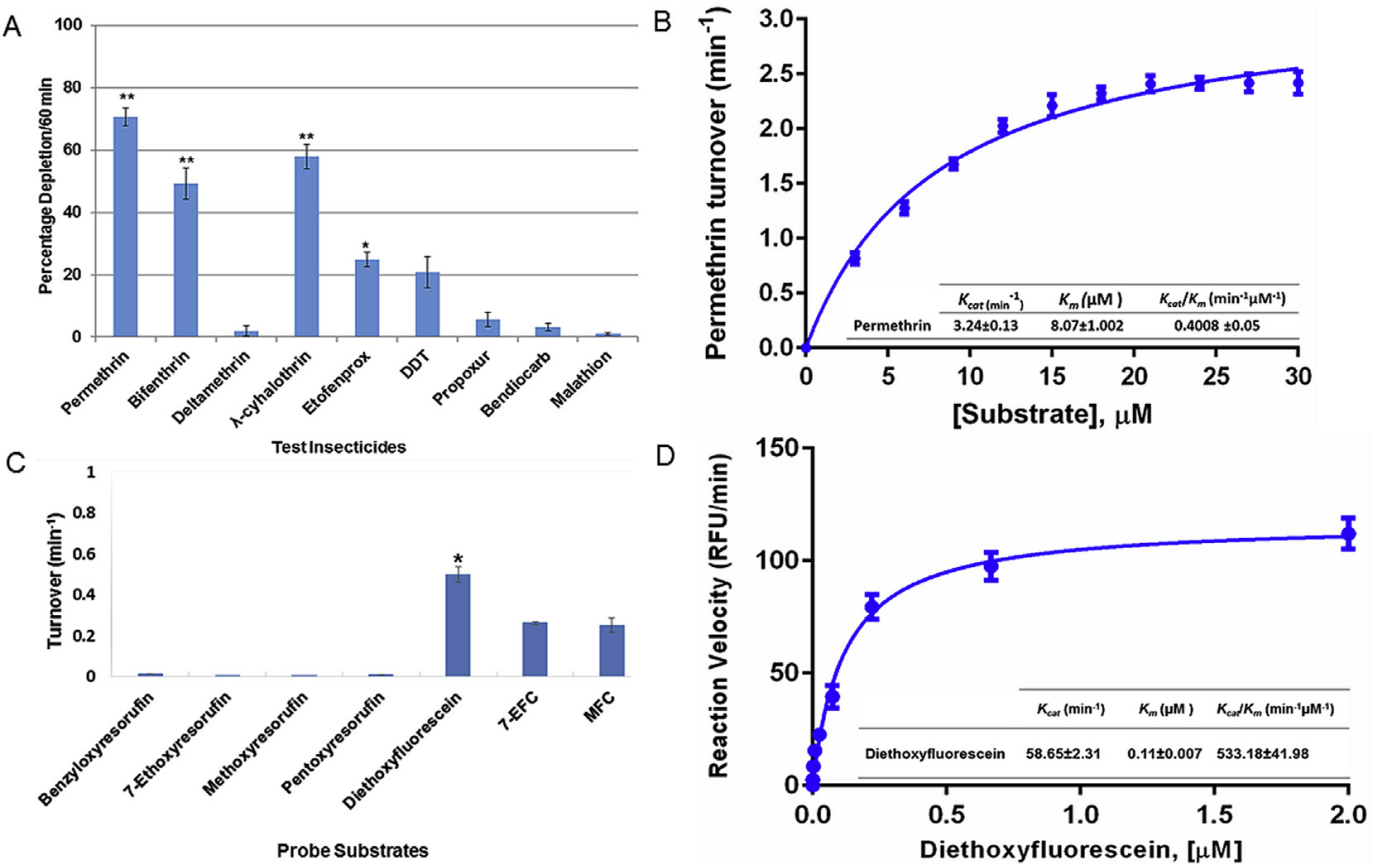
**Metabolism of insecticides and probe substrates by recombinant CYP6P4**. (A) Percentage depletion of 20 μM insecticides following incubation for 1hr. Results are mean ± S.E.M. Significantly different from negative control (without NADPH) at **p < 0.01 and *p < 0.05; (B) Michaelis-Menten plot of permethrin metabolism. Each point (n = 3) is mean ± S.D. of calculated velocity with different concentration of permethrin. Inset kinetic parameters and catalytic efficiency of permethrin metabolism; (C) Metabolism of probe substrates. The solid bars indicate average of significant turnovers of three experimental replicates compared to negative controls (-NADPH). *p < 0.05; (D) Michaelis-Menten plots of CYP6P4 metabolism of diethoxyfluorescein. Each point is a mean ± S.D. of turnover of DEF compared with negative control (-NADPH).

Recombinant CYP6P4 metabolizes pyrethroids permethrin and λ-cyhalothrin representative Type I and Type II pyrethroids respectively with high depletion of 70.5% ± 2.85 (p < 0.01) and 57.8% ± 3.88 (p < 0.01), after an hour of incubation (Fig. 1A). The enzyme also depletes substantial amount of bifenthrin (49.2% ± 5.12, p < 0.01) and the pseudo-pyrethroid etofenprox (24.9% ± 2.3, p < 0.05). However, no activity was observed toward deltamethrin (a Type II pyrethroid), with depletion of less than 2% over the course of three separate experiments. No activities were also observed with bendiocarb, propoxur and malathion. For DDT, a depletion of up to 20% ± 5.03 (p = 0.08) was obtained following addition of sodium cholate. However, neither dicofol nor DDE was detected to confirm DDT metabolism.

Steady-state kinetic parameters were established with permethrin. Metabolism of this Type I pyrethroid proceeds via Michaelis–Menten mechanism (Fig. 1B) with moderately low *K*_*m*_ (8.07 μM ± 1.002) and maximal catalytic activity (*K*_*cat*_) of 3.24 min^−1^ ± 0.13, resulting in catalytic efficiency toward permethrin of 0.4008 min^−1^μM^−1^ ± 0.05.

### 3.3 *An. arabiensis* CYP6P4 metabolism of probe substrates

Recombinant CYP6P4 metabolizes diethoxyfluorescein (DEF) with higher turnover than the coumarin-based probe substrates tested (Fig. 1C). However, no significant activity was observed with the four resurofin-based probes. To establish steady–state parameters kinetic analysis was conducted with DEF. Metabolism of DEF follows Michaelis–Menten fashion (Fig. 1D) with a high affinity (very low *K*_*m*_; 0.11 μM ± 0.0075) and moderate maximal catalytic activity (*K*_*cat*_ = 58.65 min^−1^ ± 2.31). The catalytic efficiency of CYP6P4 for DEF was thus calculated as 533.18 min^−1^μM^−1^ ± 41.98.

### 3.4 Inhibition assay

**Fig. S1:**
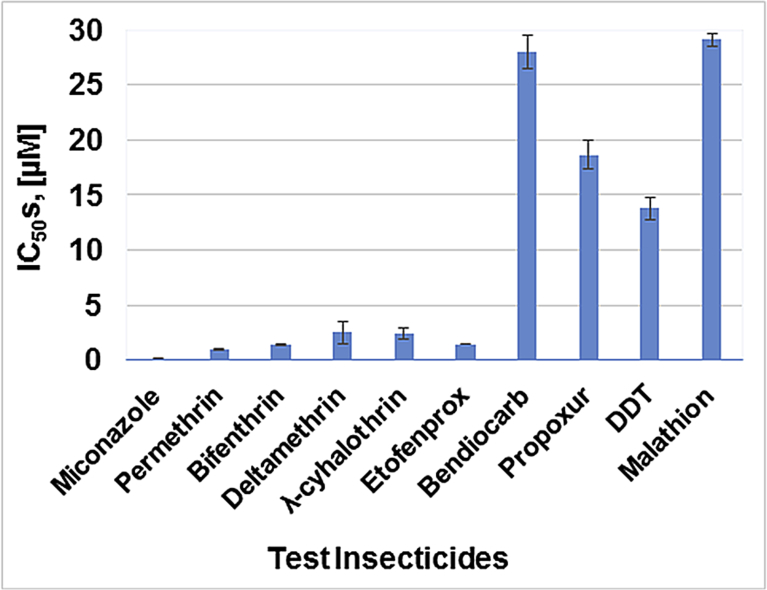
**Pattern of inhibition of CYP6P4 metabolism of diethoxyfluorescein metabolism by insecticides**. Mean IC_50_ values of the test insecticide inhibitors. Data represent mean IC_50_ at eight concentrations of each insecticide plus or minus standard deviation. Error bars variation in the values of the IC_50_ between different concentrations.

Inhibition assay revealed that the recombinant CYP6P4 binds all Type I and Type II pyrethroids with the highest degree of binding obtained with Type I pyrethroids, especially permethrin (IC_50_ = 0.97 μM ± 0.05) (Supplementary Fig. S1). Surprisingly, deltamethrin (not metabolised by CYP6P4) shows a high degree of binding (IC_50_ = 2.52 μM ± 1.002) comparable to values obtained from λ-cyhalothrin. Overall, the IC_50_s obtained from all the pyrethroids indicate good binding to CYP6P4. In contrast, high IC_50_ values were obtained with insecticides from other classes, especially malathion and bendiocarb with the highest IC_50_s of all insecticides tested in line with the full susceptibility observed to these insecticides by *An. arabiensis*.

### 3.5 In silico prediction of insecticides binding parameters and conformation

In order to understand the underlying mechanism which causes this P450 to metabolize all pyrethroids except deltamethrin, molecular docking simulation was conducted using the homology model of the P450 with insecticides from various classes. In addition, non-pyrethroids including bendiocarb, DDT and malathion were also docked. The binding parameters for each insecticide are given in Supplementary Table S2, while the top ranked predicted score for productive pose of each insecticide, binding mode and expected site of metabolism are summarised in Table 1. Type I pyrethroids and etofenprox exhibited the highest score consistent with the lowest IC_50_ values obtained with permethrin and bifenthrin. The Type II pyrethroid λ-cyhalothrin exhibited the high score as consistent with the depletion it exhibited from metabolism assays. In contrast, deltamethrin exhibited good score also consistent with the low IC_50_ obtained, though no metabolism was observed with it.

**Fig. 2:**
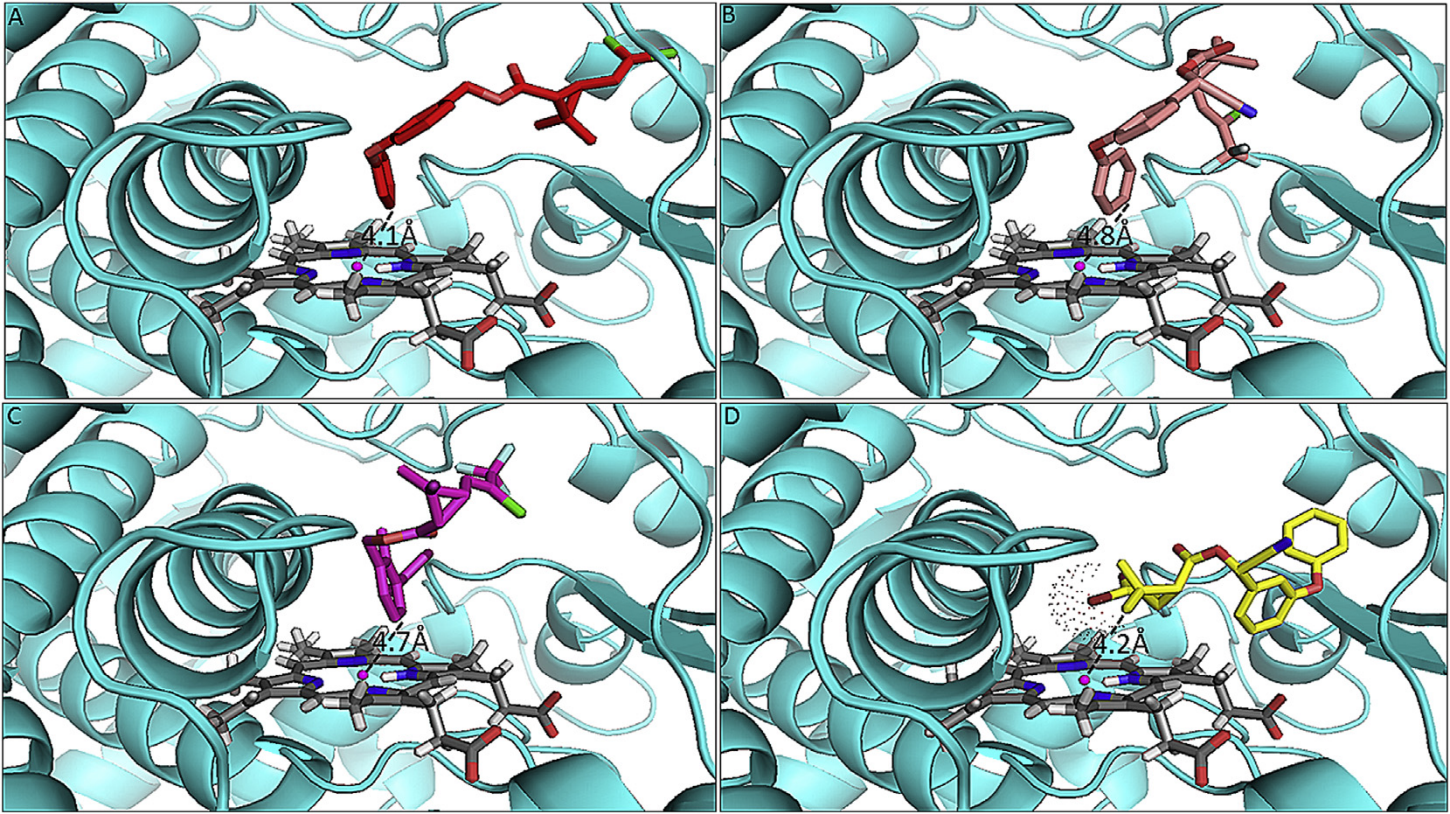
**Predicted binding mode of (A) permethrin (red stick), (B) λ-cyhalothrin (dirty violet), (C) bifenthrin (magenta), and (D) deltamethrin (yellow)**. CYP6P4 helices are presented in cyan colour; heme atoms are in stick format and grey. Distance between possible sites of metabolism and heme iron are annotated in Angstrom. Note the bromine atom of dihalovinyl moiety of deltamethrin are in red and dotted.

**Fig. 3:**
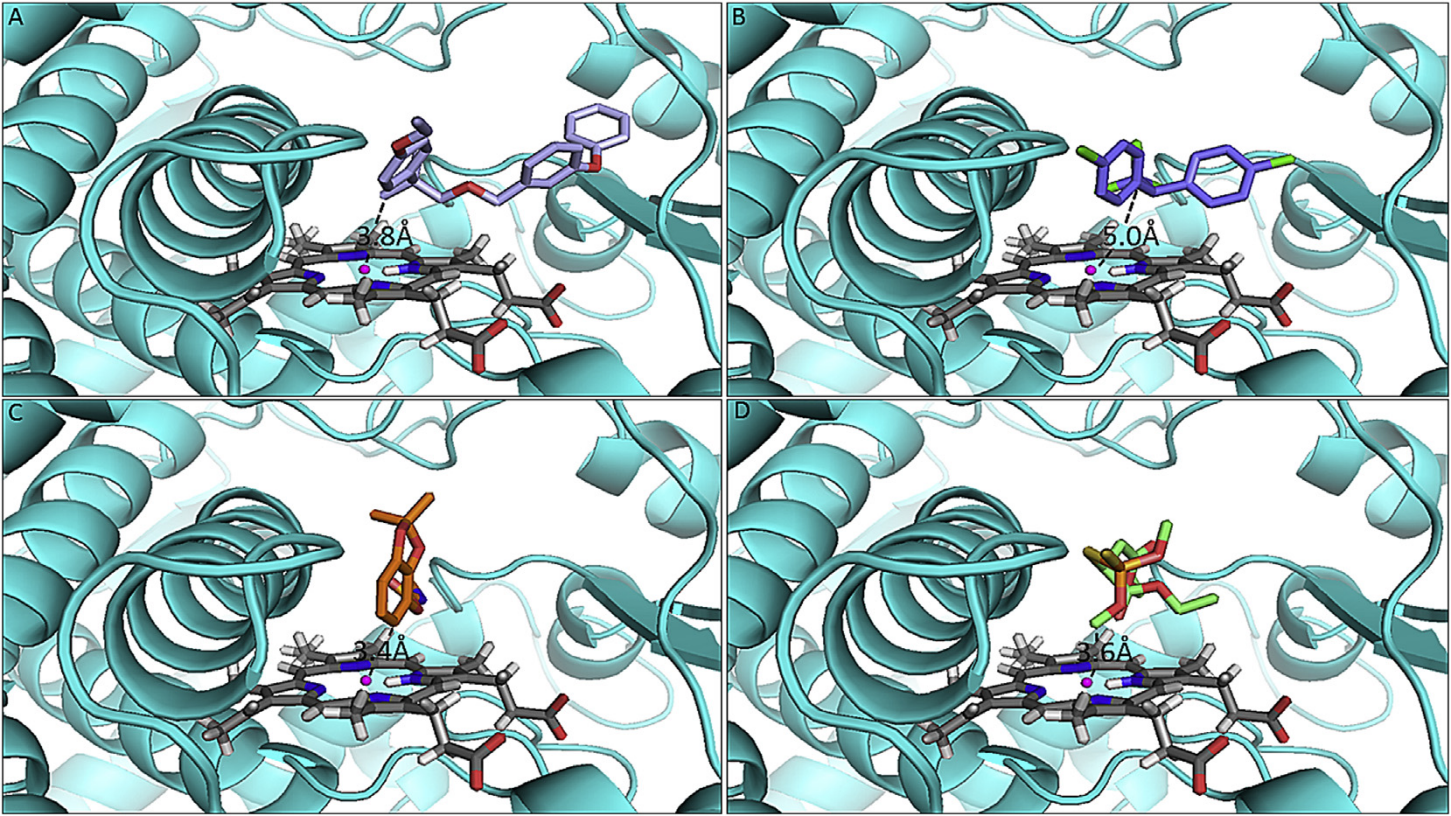
**Predicted binding mode of (A) etofenprox (light-blue stick), (B) DDT (purple-blue), (C) bendiocarb (orange), and (D) malathion (green stick)**. CYP6P4 helices are presented in cyan colour; heme atoms are in stick format and grey. Distance between possible sites of metabolism and heme iron are annotated in Angstrom.

Comparison of binding conformations of the insecticides in the active site of the CYP6P4 model revealed the possible molecular mechanism through which this P450 binds and metabolises Type I and Type II pyrethroids with the exception of deltamethrin. Permethrin and λ-cyhalothrin, the two insecticides with the highest depletion, docked with the phenoxy ring oriented above the heme and carbon 4′ located 4.1 Å and 4.8 Å from heme iron respectively (Fig. 2A and B). This posture leads to 4′-hydroxy metabolite and has been described as the major route of metabolism of some pyrethroids; for example in *An. gambiae CYP6M2* (Stevenson et al., 2011) and in other organisms (Gilbert and Gill, 2010). Bifenthrin and etofenprox also docked productively, with C6 of the benzyl ring above the heme for bifenthrin, and one of the methyl groups of 2-methylpropoxy moiety above the heme for etofenprox (Fig. 2, Fig. 3A, respectively). Though DDT produced a good binding score, the organochloride docked potentially unproductively (Fig. 3B) with a trichloromethyl group approaching the heme, and the chlorine atoms projected above the heme catalytic centre. A productive pose of DDT has been established with C-1 of the trichloromethyl group docked above the heme in *An. gambiae CYP6Z1* (Chiu et al., 2008).

Of all the pyrethroids, only deltamethrin docked differently with the gem dimethyl groups of the cyclopropane moiety approaching the heme (Fig. 2D) in the top 11 ranked solutions. In this posture hydroxylation to generate *cis* or *trans*-methyl deltamethrin is possible, however this mode has been described as a minor route of metabolism in insect P450s (Stevenson et al., 2011). In addition, the insecticide docked with the bromine atoms of the dihalovinyl moiety positioned directly above the heme; this possibly hinders the intermolecular interactions required for metabolism to take place.

**Fig. S2:**
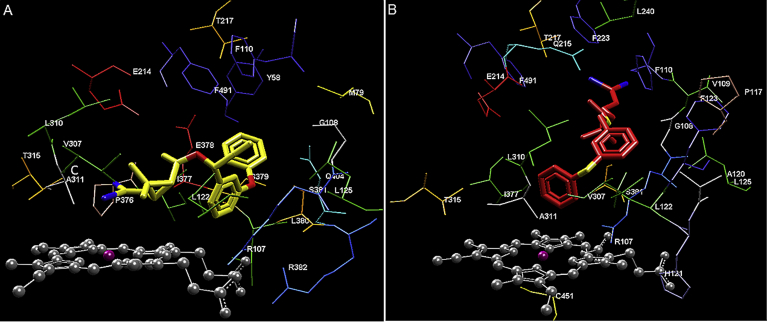
**Binding mode of deltamethrin and permethrin in CYP6P4 model**. (A and B) Residues predicted to be within 5.0 Å of deltamethrin (yellow stick) and permethrin (red stick), respectively.

To further understand the intermolecular interactions between pyrethroids and model of CYP6P4, comparative analysis of the docking solution for deltamethrin and permethrin was carried out using the Molegro Molecular Viewer. Within 5.0 Å radius both deltamethrin and permethrin were surrounded by the same type of amino acid residues (Supplementary Figures 2A and 2B respectively), but the former insecticide docked with acid moiety approaching the heme, with aromatic residues possibly stabilizing the aromatic rings away from heme catalytic centre through resonance stabilisation and *π*-stacking. In the case of permethrin the aromatic residues of CYP6P4 possibly bind the acid moiety allowing the alcohol group to approach the heme, enhancing optimal interaction and metabolism. Also, in contrast with the pose of permethrin, the bromine atoms of deltamethrin were within 3.3 Å of residue Pro^376^. Possibly this results in steric hindrance due to vdW overlaps between the bromine atoms and the prolyl side chain, which obstructs deltamethrin from approaching closer to the heme.

**Fig. S3:**
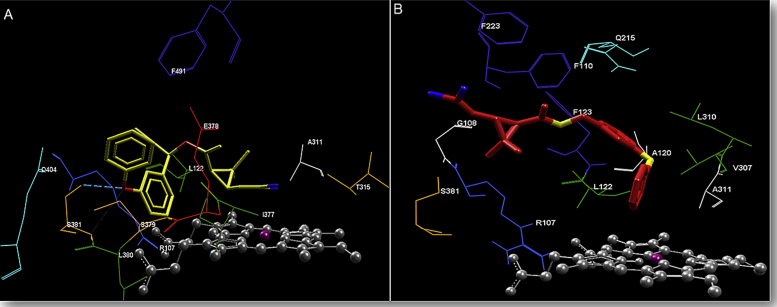
**Intermolecular hydrogen bonds between pyrethroid structures and CYP6P4 active site residues**. (A) Two intermolecular hydrogen bonds were predicted between deltamethrin and CYP6P4 residues. (D) Note the absence of hydrogen bonds between permethrin and surrounding amino acids in the active site of CYP6P4.

Two intermolecular hydrogen bonds were also predicted for deltamethrin (Supplementary Figure 3A): (i) donated by the alcohol side chain of Ser^381^ to phenoxy oxygen, contributing an energy of −2.5 kJ/mol, within a distance of 2.85 Å; (ii) donated by the peptide bond between Leu^380^ and Ser^381^ to phenoxy oxygen, with an energy of −0.24 kJ/mol and distance of 3.08 Å. It is predicted that these hydrogen bonds further stabilised deltamethrin away from the heme catalytic centre. Despite the presence of similar amino acid residues in the binding site of permethrin, no intermolecular hydrogen bonds were predicted for this insecticide and CYP6P4 active site residues (Supplementary Figure 3B).

Bendiocarb docked with C-6 of the aromatic ring oriented above the heme iron (ring hydroxylation to 5- or 6-hydroxybendiocarb was predicted) (Fig. 3C), but no metabolism was observed with bendiocarb indicating that hydroxylation of this carbamate may not proceed via ring hydroxylation. Malathion docked with the methyl group of dimethylphosphate above the heme iron within a distance of 3.6 Å (Fig. 3D). In this posture demethylation to desmethylmalathion is possible, but no metabolism of malathion was observed as well, from *in vitro* assays. The thiol moiety of the thiophosphate is oriented away from the heme catalytic centre suggesting a lack of oxidative desulfuration mechanism through which P450s generates oxon analogues (Krieger, 2010).

**Fig. 4:**
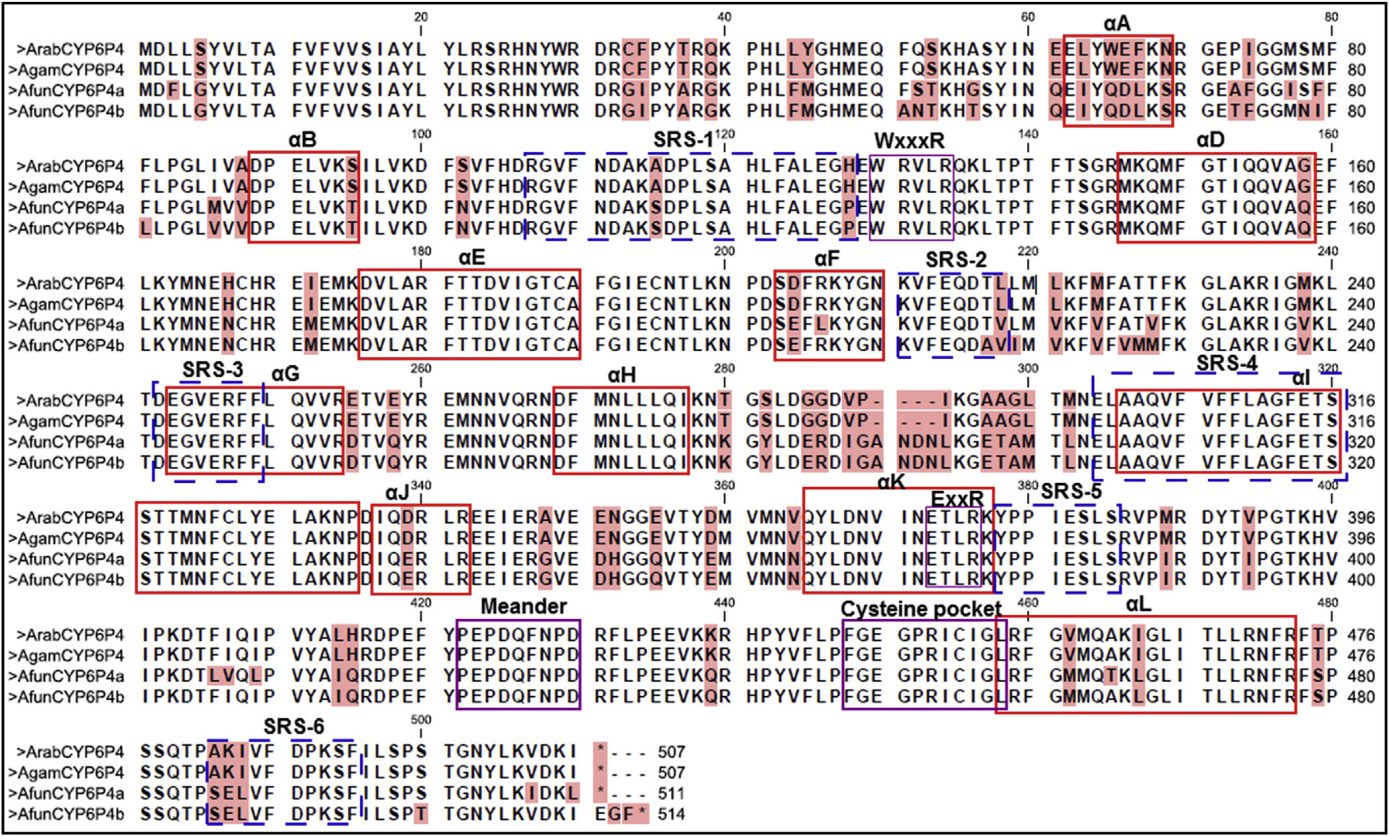
**Comparison of *An. arabiensis CYP6P4* amino acid sequences with orthologs from *An. gambiae* and *An. funestus***. The solid, red lines represent helices A-L, while dashed blue lines correspond to the substrate recognition sites 1–6. Solid purple lines identified the structurally conserved motifs of the P450s. Variable residues are highlighted in pink. (For interpretation of the references to colour in this figure legend, the reader is referred to the web version of this article.)

Sequence characterisation of *An. arabiensis CYP6P4* indicates that it is identical with its ortholog from *An. gambiae* (AGAP002867-RA) (Fig. 4) with 506 amino acids each. The conservation of this gene could suggest that its detoxification function was retained even after speciation. Of course, this gene has been recently described among a handful of P450s responsible for pyrethroid resistance in field populations of *An. coluzzii* and *An. gambiae* from other neighbour countries in West Africa (Edi et al., 2014, Toe et al., 2015). The gene shares 85% and 86% identity respectively with *CYP6P4a* (510 amino acids) and *CYP6P4b* (513 amino acids) from the other major malaria vector, *An. funestus* (Wondji et al., 2009); the two duplicated P450s from *rp1* QTL implicated in pyrethroid resistance.

Not surprising, the WxxxR motif, the signatory oxygen-binding pocket (AGFETS)/proton transfer groove, the characteristic ExxR motif which stabilizes the heme structural core, the cysteine pocket/heme-binding region (PFxxGxxxCxG), which forms the fifth axial ligand to the heme iron, and the ‘*meander*’ (Feyereisen, 2012, Werck-Reichhart and Feyereisen, 2000) were all identical and conserved in the four different sequences (Fig. 4). In terms of sequence-activity relationship, variations which could impact activity of *An. arabiensis*/*An. gambiae CYP6P4* compared with *An. funestus CYP6P4a/b* were observed in the BC loop within the SRS-1 (Ala^115^Ser and His^128^Pro) and three amino acid differences in the NH_2_-terminus of SRS-6. Another major difference was also observed in the composition of the residues linking αH to the NH_2_-terminus of I helix and within the αA helix.

## 4 Discussion

The effectiveness of insecticide-based control of mosquito vectors depends on several factors, including the composition of mosquito species in a reference setting and the susceptibility of the mosquito population. Given the heterogeneity in patterns of insecticide resistance even within the same localities in Africa, a detailed knowledge of the characteristics of the resistance and its molecular basis is a prerequisite for timely implementation of the suitable control tools. *An. arabiensis* is an important malaria vector and its control is of high priority particularly as its outdoor biting and feeding flexibility can sustain malaria transmission even in the presence of indoor-based control interventions such as LLINs and IRS (Griffin et al., 2010). This study has dissected the molecular basis of the P450-mediated resistance establishing that the previously reported over-expressed P450 *CYP6P4* (Witzig et al., 2013) is responsible for the pyrethroid resistance.

*An. arabiensis CYP6P4* is an important pyrethroids metaboliser which detoxifies insecticides used for impregnation of bed nets and for IRS. Highest activity was obtained with permethrin which exhibited catalytic activity half the value established for *An. gambiae CYP6M2* (Stevenson et al., 2011) but with comparable catalytic efficiencies due to higher affinity exhibited by *CYP6P4*. These catalytic efficiencies of *An. arabiensis CYP6P4* with permethrin were also similar to the values we have established for the *An. funestus CYP6P9a* and *CYP6M7* resistance genes (Riveron et al., 2014). With the exception of DDT results from metabolism assays with recombinant *CYP6P4* are in line with the insecticides resistance profile observed in the *Ndja* populations (Witzig et al., 2013). In the absence of over-expression of *CYP6M2*-a P450 established to confer DDT resistance in *An. gambiae* (Mitchell et al., 2012), a yet uncharacterised mechanism conferring DDT resistance cannot be ruled out.

*CYP6P4* cannot metabolise bendiocarb and malathion; these and the absence of *ace1* mutation in the *Ndja* population (Witzig et al., 2013) probably explain the susceptibility of the populations to carbamate and organophosphate insecticides. *CYP6P4* could be the driver of pyrethroid resistance in this *Ndja* population and possibly *An. arabiensis* populations from neighbouring regions. This is because qRT-PCR analysis conducted by Witzig and colleagues (Witzig et al., 2013) revealed that the two candidate genes *CYP6P3* (from the *rp1* QTL) and *CYP6M2* established to confer cross-resistance to the sister species *An. gambiae* were not over-expressed in the *An. arabiensis* from *Ndja*. It is necessary to study this gene further in order to identify potential, beneficial polymorphisms associated with its resistance, such as markers that could pave ways to establish diagnostic tools. Further studies with populations of *An. arabiensis* from this region need to be carried out in order to find out other metabolic resistance genes or yet uncharacterised target-site insensitivity mutations which could confer the marginal resistance to deltamethrin.

Molecular docking simulations are increasingly used to characterise P450 insecticide metabolisers and to rationalise the heterogeneities observed in terms of substrate preferences and metabolism (Chandor-Proust et al., 2013, McLaughlin et al., 2008, Stevenson et al., 2011). For example, *An. gambiae CYP6Z2* has been established as non-metaboliser of permethrin and cypermethrin though it binds these insecticides (McLaughlin et al., 2008), just like the observation we made with recombinant CYP6P4 from *An. arabiensis*. Not surprisingly, deltamethrin docked into the active site of the CYP6P4 model in the same unproductive pose that permethrin and cypermethrin bind to the model of CYP6Z2 in the above study. Additionally some P450s possess arrays of insecticides to metabolise and closely related P450s can be restricted to a narrow range of chemicals to detoxify (Schuler and Berenbaum, 2013), but a particular P450 may have a limited range of substrates even among insecticides of the same class. For example, *CYP6P7* from *Anopheles minimus* is known to metabolise and confer resistance to permethrin, deltamethrin and cypermethrin, but is incapable of metabolising λ-cyhalothrin (Duangkaew et al., 2011). This was attributed to narrow channel opening to the heme iron which constrains access for this λ-cyhalothrin into the heme catalytic hotspot (Lertkiatmongkol et al., 2011).

The inability of *CYP6P4* to metabolise deltamethrin, and other non-pyrethroid insecticides provide opportunity for evidence-based management of resistance. It shows that the heterogeneity of resistance to insecticides from the same chemical class should be taken into account in designing resistance management. However, this can only be applicable with consistent monitoring of the type and cause(s) of resistance in the different sub-regions. For example, while *kdr* was recorded as absent in *An. arabiensis* populations from Niger, Chad and northern Cameroon (Czeher et al., 2008, Nwane et al., 2013, Nwane et al., 2011, Witzig et al., 2013), the 1014F mutation has been described in populations from neighbouring Sudan (Abdalla et al., 2014) and Burkina Faso (Dabire et al., 2014). Also, we have recently discovered both 1014F and 1014S mutations in *An. arabiensis* populations from Sudan savannah of northern Nigeria, albeit in low frequency (Ibrahim et al., 2014). This contrasting pattern in *kdr* frequency may also extend to metabolic resistance; thus caution should be exercised when deploying malaria control tools. The contribution of other sympatric Anopheles species e.g. *An. coluzzii*/*gambiae* and *An. funestus*, towards malaria transmission in this region should also be investigated and taken into account. At this moment malaria burden can only be curbed through diligent monitoring, timely implementation of suitable control tools and evidence-based management of insecticide resistance.

## 5 Conclusion

Knowledge of resistance and its underlying mechanisms is essential to management of insecticide resistance in malarial mosquitoes. However, such knowledge requires a thorough understanding of the molecular basis of the resistance in order to inform control programs on the right tools to effectively manage the resistance. Here, we established that the P450 *CYP6P4* is responsible for resistance against the bed net insecticide (permethrin) in Sudano-Sahelian populations of *An. arabiensis* from Central Africa. It is hoped that this information can help guide the National Malaria Control Program in choosing the right control tools to be used in the region. However, there is a need to find out the current resistance profiles of *An. arabiensis* population from this region in order to identify temporal changes which might have occurred since collection of the populations used in this study.

## Accession numbers

The DNA sequence of *CYP6P4* reported in this paper have been deposited in the GenBank database (Accession # KT698165).

## Conflicts of interest

The authors declare no competing interests.

## Authors' contribution

CSW conceived and designed the study. SSI performed the molecular and biochemical experiments with contribution from JMR, RS and HI; SSI analysed the data with contribution from CSW and JMR; SSI and CSW wrote the manuscript with contribution from all authors.

## Figures and Tables

**Table 1:** Predicted binding score and probable site of metabolism of various insecticides by CYP6P4.

Insecticide	Structure	PLANTS_PLP_ score	Distance to heme iron (Å)	Predicted site of metabolism
1*R*-*cis* permethrin ZINC01850376		- 72.99	4.1	4′ spot of the phenoxy ring
Bifenthrin ZINC02516821		−79.06	4.7	C-6 of the benzyl ring
Deltamethrin ZINC01997854		−68.09	4.2	*trans*-methyl group of cyclopropane
λ-cyhalothrin ZINC013827939		−71.25	4.8	4′ spot of the phenoxy ring
Etofenprox ZINC02558051		−70.16	3.8	-CH_3_ of 2-methylpropoxy moiety
Bendiocarb ZINC02015426		−45.74	3.4	C5/6 of the aromatic ring
DDT ZINC01530011		−57.30	5.0	Trichloromethyl group
Malathion ZINC01530799		−42.81	3.6	-CH_3_ of dimethyl thiophosphate

## Appendix A Supplementary data

Table S1 List of primers used in this study.

Table S2 Predicted binding parameters of the top-ranked docking mode of various insecticides in CYP6P4 model.
