# Supplementary material for: The cytochrome P450 CYP6P4 is responsible for the high pyrethroid resistance in knockdown resistance-free Anopheles arabiensis
Source: Insect Biochem Mol Biol. 2016 Jan;68:23–32. doi: 10.1016/j.ibmb.2015.10.015 (PMC4717123; doi:10.1016/j.ibmb.2015.10.015)
Supplement: Table S1 — List of primers used in this study. [file mmc1.docx]

| Gene/Allele | Forward Primer | Reverse Primer |  |
| --- | --- | --- | --- |
| Primers used for amplification of *CYP6P4* and *in vitro* functional characterisation | | | |
| ArabCYP6P4_Full | ATAGATCTATGGATCTGTTAAGCTACGTG | TCTCTAGACTATATCTTATCAACCTTCAGAT |  |
| ompA+2 Forward | GGAATTCCATATGAAAAAGACAGCTATCGCG |  |  |
| ompA+2 CYP6P4 Forward | CGTAGCTTAACAGATCCATCGGAGCGGCCTGCGCTACGGTAGCGAA | |  |
| ompA+2 CYP6P4Reverse |  | TCTAGAGAATTC CATTGATAACGTTGTCCAGA |  |

Blue is *Bgl*II, Green is *EcoR*I, Purple is *Nde*I and Red is *Xba*I. For ompA+2 CYP6P4 Forward, the last 21 bases linker of the leader sequence is underlined.
