# Supplementary material for: The cytochrome P450 CYP6P4 is responsible for the high pyrethroid resistance in knockdown resistance-free Anopheles arabiensis
Source: Insect Biochem Mol Biol. 2016 Jan;68:23–32. doi: 10.1016/j.ibmb.2015.10.015 (PMC4717123; doi:10.1016/j.ibmb.2015.10.015)
Supplement: Table S2 — Predicted binding parameters of the top-ranked docking mode of various insecticides in CYP6P4 model. [file mmc2.docx]

| Insecticide | Rank | Atoms | Weight | PLANTS_PLP_ Score | Flexible bonds | Hydrogen bond score | Metal interaction score | Steric interaction score | Ligand conformation penalty | RMSD (Å) |
| --- | --- | --- | --- | --- | --- | --- | --- | --- | --- | --- |
| Permethrin | 1^st^ | 46 | 391.29 | -72.99 | 7 | 0.00 | 0.00 | -74.07 | 5.16 | 29.08 |
| Bifenthrin | 1^st^ | 51 | 422.87 | -79.06 | 9 | 0.00 | 0.00 | -82.47 | 5.41 | 25.75 |
| Deltamethrin | 1^st^ | 47 | 505.21 | -68.90 | 8 | -2.00 | 0.00 | -80.66 | 11.67 | 30.57 |
| λ-cyhalothrin | 1^st^ | 50 | 449.85 | -71.25 | 9 | -0.48 | 0.00 | -78.06 | 8.02 | 30.63 |
| Etofenprox | 1^st^ | 56 | 376.50 | -70.16 | 9 | -2.00 | 0.00 | -82.47 | 5.41 | 25.75 |
| Bendiocarb | 1^st^ | 29 | 223.23 | -45.74 | 2 | -0.98 | 0.00 | -46.60 | 1.84 | 30.96 |
| DDT | 1^st^ | 28 | 354.48 | -57.30 | 3 | 0.00 | 0.00 | -58.07 | 0.78 | 30.45 |
| Malathion | 1^st^ | 38 | 330.35 | -42.81 | 11 | 0.00 | 0.00 | -44.03 | 3.22 | 25.59 |

PLANTS_PLP_ = *Piece-wise linear potential* Protein-Ligand ANT System, and RMSD = Root mean square deviation.
